# Journal of Cardiovascular Magnetic Resonance: Open access in 2008

**DOI:** 10.1186/1532-429X-10-1

**Published:** 2008-01-15

**Authors:** Dudley J Pennell

**Affiliations:** 1 Imperial College and Royal Brompton Hospital, London, UK

## 

This is the first article in the open access *Journal of Cardiovascular Magnetic Resonance *which will be published exclusively on-line from January 2008 and available on [1]. We have now made the transition from the traditional publication system and look forward to the advantages of the internet era. This change has been made possible by a large number of people, and in particular I would like to thank the Publications Committee and Board of the Society for Cardiovascular Magnetic Resonance and the journal editorial team, who have all given their full support. I have written about this change in the closing editorials of the 2007 paper-based issues with our previous publisher [2], but I feel it is important to once again summarise the reasons for the change and the new procedures, so that they are firmly recorded at the outset of this new venture with our new publisher BioMed Central (BMC). The full range of BMC open access journals can be viewed online [3].

## Why open access?

The traditional publication method has the disadvantage of research not being available to many without charge. This limits its visibility and impact. There is evidence that open access journal articles are more frequently accessed, which results in a greater impact and a higher citation rate [4]. Other attractive features for researchers include ownership of the copyright to their papers, same day posting of the manuscript on acceptance (in provisional form, with replacement by the final PDF and HTML versions within a few weeks after XML mark-up and typesetting), free use of colour, and the possibility of embedding supplementary material directly within the primary article (for example cines, data, weblinks) at no extra cost. One disadvantage of the new model is that no paper version of JCMR will be produced, but we believe this is more than offset by rapid email notification of new articles going online (please sign up for article alerts [1]) and direct selective printing in the office where needed, which disseminates articles faster with reduced environmental impact. Clearly, open access also means that research is available in economically disadvantaged regions of the world, and to the public – who have often paid for the research in the first place. Table 1 compares the publishing models, and a number of references are listed below which document some of the reports and discussions regarding the adoption of open access [5-9].

**Table 1:** Comparisons between the publishing models

	*Conventional Publishing*	*Open Access Publishing*
History of basic principles	First printing press made by Johannes Gutenberg in 1440. Many printing companies worldwide. Significant recent consolidation.	Internet first working in 1969. PDF files mimic printed page, and prevent alteration of content. BioMed Central publishes open access articles from 2000.
Copyright ownership	Publisher	Author
Payment for publishing articles	Usually none, but some journals have a "per page" charge	The institution, research funder, or author submitting the article
Payment for reading articles	The readers through payment or subscriptions	None
Submission	Usually internet	Internet
Publication	Usually paper and internet	Internet
Peer review	Yes	Yes
Time to publication after acceptance	Highly variable, from same day posting of manuscript, to many months later in print	Same day posting of manuscript with replacement by typeset final version some weeks later
Visibility	Variable, other than for the abstract, and depends on cost of access	High, with free full article access
Number of articles	Limited by issue/volume pages	Unlimited
Typical proponents	Publishers	Public and charitable funders: eg National Institutes of Health (USA), Wellcome Trust (UK), Medical Research Council (UK), Howard Hughes Medical Institute (USA)
Cost of use of colour	$500–1000 per printed page	None
Paper copy in hand	Yes	Only by printing the article
Embedding of cines	Not on paper	Yes, in the primary article
Citation index	Yes	Yes
Cited by PubMed and other scholarly search sites	Yes	Yes
Trend in citation index	Neutral	Increase through visibility
Long term archiving	Yes	Yes
Back archive	Yes	Yes

## Manuscript submission procedures

For researchers, the new manuscript submission system can be accessed on-line [1] and is now fully operational. Please submit all new manuscripts through this system. The system is very similar to manuscript submission operated by other major journals. There may be some minor teething problems with its use in the first months, mainly because the editorial team has to adjust to using it, but we anticipate the impact of this learning curve will be very limited.

## The article processing charge (APC)

The system of paying for the journal is very different from the conventional subscription model. In order that the costs of producing, publishing and maintaining the papers online can be met, each article accepted for publication is charged an article processing charge (APC). This charge is agreed between SCMR and BMC by contract and is £650 (approximately $1300) for 2008. Authors must indicate during the manuscript submission how the APC will be charged (see below) should their paper be accepted. There are several routes of APC payment:

• Many institutions have "full" BMC membership so that the full APC is covered on behalf of authors at the time of submission (often automatically) and later claimed by BMC direct from the institution. Examples of full institutional membership include the National Health Service (NHS) in the UK, and the Cleveland Clinic in the USA. A list of full and supporter institutional members of BMC is available on-line [10]. There is a verification process for institutional APC payment, which includes automatic recognition of IP address, confirmation of a qualifying email domain, or if necessary, by email communication with the customer services department at BMC.

• Some institutions have "supporter" memberships, meaning that they cover a proportion of the APC cost. The research team is then responsible for the remainder.

• Researchers are now being encouraged to add the expected APC costs for anticipated articles to grant applications, and increasingly therefore, the APC might be paid direct from dedicated lines on research grants.

• For SCMR members, SCMR has offered to cover the APC cost for the current time. This period will be regularly assessed by the board.

• Researchers without the financial means to pay the APC can apply direct to BMC for an "APC waiver", which is granted at the publisher's sole discretion.

• Failing all the above, the research team is responsible for paying the APC.

## Your editorial team

The journal editorial team is based in London UK, at Royal Brompton Hospital. We are fortunate to have a highly experienced and critical mass of Assistant Editors in David Firmin, Raad Mohiaddin, Philip Kilner and Sanjay Prasad who take primary responsibility for guiding the manuscripts through the peer-review process. Warren Manning (Deputy Editor, Boston, USA) and Stefan Neubauer (Assistant Editor, Oxford, UK) take responsibility for articles requiring special expertise or additional opinions, and where conflicts of interest arise for the Royal Brompton editorial team. All final decisions regarding publication are made by the Editor in Chief at weekly hanging committee meetings.

## Managing Editor

This administrative position is crucial to the operating efficiency of the journal. We are fortunate to have Ms Fei Wang in this post, and all enquiries regarding the journal should be made in the first instance to her on JCMR@ic.ac.uk.

## Editorial board changes

I have undertaken a thorough review of the editorial board in 2007, and after consultation, I have refreshed the board, and it now has approximately 50% new members. I would like to thank the editorial board members who I have asked to stand down for their long and helpful service to the journal. I am grateful for the many kind and understanding comments from those standing down and for their gracious acceptance that renewal is important, and I am certain that I will still be able to call upon them in special circumstances for their advice. The role of the editorial board is to advise the Editor on policy, and also to commit to review of manuscripts. We will now adopt the principle of review of the editorial board every 2 years for rather more frequent but less radical change. The new editorial board is available for review on-line [1].

## Access to previously published articles

JCMR articles published in the first 9 volumes are available on-line [11].

## Summary

Taking JCMR into the open access arena is a challenge, but presents many opportunities. We believe that the move has many advantages, and is well suited to an imaging journal which routinely uses high quality pictures, cines and colour. We hope you will support us in this endeavour. In return, we remain committed to providing the CMR community with a high-quality portal for new research findings which is fast to publication, impactful, and available to the widest possible audience.

## References

[B1] Journal of Cardiovascular Resonance. http://www.jcmr-online.com/.

[B2] Pennell D (2007). New Publishing Arrangements for JCMR. Journal of Cardiovascular Magnetic Resonance.

[B3] BioMed Central. http://www.biomedcentral.com/.

[B4] Eysenbach G Citation Advantage of Open Access Articles. PLoS Biology.

[B5] Gass A, Doyle H (2005). The reality of open-access journal articles. The Chronicle of Higher Education The Chronicle Review.

[B6] Scientific publications: Free for all? House of Commons Science and Technology Committee. Tenth report of session 2003–4. http://www.publications.parliament.uk/pa/cm200304/cmselect/cmsctech/399/399.pdf.

[B7] Open Access Myths. http://www.biomedcentral.com/openaccess/inquiry/myths/.

[B8] Dotinga R Open Access Launches Journal Wars. Wired.

[B9] Giles J PR's 'pit bull' takes on open access. Nature.

[B10] BioMed Central Institutional Members Page. http://www.biomedcentral.com/inst/.

[B11] Informa's home page for Journal of Cardiovascular Resonance. http://www.informaworld.com/smpp/title~content=t713597265~tab=issueslist.

